# Assessment of Some Trace Metals in Head and Neck Squamous Cell Carcinoma: A Case-Control Study 

**DOI:** 10.30476/dentjods.2025.104402.2534

**Published:** 2025-12-01

**Authors:** Zohreh Dalirsani, Zahra Delavarian, Atessa Pakfetrat, Seyed Isaac Hashemy, Leyla Vazifeh Mostaan, Fahimeh Khaghani, Mahboobeh Taherizadeh, Maede Shokri, Hossein Alavi

**Affiliations:** 1 Oral and Maxillofacial Diseases Research Center, Mashhad University of Medical Sciences, Mashhad, Iran.; 2 Dept. of Clinical Biochemistry, Faculty of Medicine, Mashhad University of Medical Sciences, Mashhad, Iran.; 3 Dept. of Otolaryngology, Cancer Research Center, Mashhad University of Medical Sciences, Mashhad, Iran.; 4 Periodontics, Private Practice, Ahwaz, Iran.; 5 Master Degree of Mathematical Statistics, Mashhad, Iran.; 6 General Dentist, Mashhad, Iran.; 7 Medical Student, Faculty of Medicine, Mashhad University of Medical Sciences, Mashhad, Iran.

**Keywords:** Micronutrients, Trace elements, Squamous cell carcinoma of head and neck, Neoplasms

## Abstract

**Background:**

Micronutrients and trace elements have been linked to the development of head and neck squamous cell carcinoma (HNSCC). However, the role of these elements in the etiology of HNSCC remains unclear.

**Purpose:**

This study was conducted to investigate the association between micronutrient and trace element levels and the risk of HNSCC.

**Materials and Method:**

In this case-control study, serum concentrations of iron, selenium, zinc, copper, and calcium were measured in 40 patients with HNSCC and compared to those of 37 healthy subjects. Statistical analysis was conducted using the Mann-Whitney test, Chi-square test, and independent t-test.

**Results:**

The mean ages of the case and control groups were 62.80±13.029 and 57.92± 9.367, respectively, with 45% of HNSCC patients and 59.5% of control subjects being male (*p*= 0.205). Serum concentrations of calcium and copper were higher, and those of selenium, zinc, and iron lower in the case group than in the control group. Adjusted logistic regression showed only copper, calcium, and iron concentrations to be significantly different between the two groups (*p*= 0.003, *p*= 0.018, and *p*= 0.047, respectively).

**Conclusion:**

The case group had lower levels of iron, zinc, and selenium, and higher levels of calcium and copper than the control group. Evaluating serum concentrations of these trace metals could be useful for further screening of individuals at high risk.

## Introduction

Head and neck squamous cell carcinoma (HNSCC) is a prevalent malignancy in adults, with varying prevalence rates among different populations. Socioeconomic status, access to healthcare, and screening services are potential risk factors for this heterogeneity [ 1 ]. Oral cancer is the most frequent type of head and neck cancer, with squamous cell carcinoma (SCC) accounting for around 90% of cases, and is predominantly observed in men aged over 60 [ 2 ].

The development of cancer is a complex and multifaceted process, which can be influenced by a range of genetic and environmental factors, such as smoking, ultraviolet radiation, and viruses. These factors can lead to alterations in the DNA of healthy cells, thereby increasing the likelihood of cancer. Furthermore, any factor that influences the proliferation, differentiation, or apoptosis of cells could impact the probability of cancer development [ 3 - 4 ].

Nutrition is essential for maintaining health and preventing diseases, including cancer. Vitamins and dietary supplements have been linked to immune system modulation and may be involved in the development of chronic illnesses. Enhancing nutritional status during cancer treatment may promote improved immune system performance and better outcomes [ 3 , 5 - 6 ].

A study conducted in Nigeria revealed a significant decrease in plasma selenium, zinc, and iron levels among patients with HNSCC compared to healthy controls [ 7 ].

Zinc, copper, and selenium are essential for numerous biochemical reactions necessary for survival. These elements also act as scavengers of free radicals, thereby slowing the cellular aging process and decreasing the risk of cancer development. However, high concentrations of copper may lead to an increase in estrogen, which may consequently be a contributing factor to the development of certain cancers [ 8 ]. Furthermore, copper is essential for the activity of several enzymes involved in maintaining oxidative balance [ 9 ] and those involved in osteoclastogenesis [ 10 ]. Iron is an essential trace metal for cells, playing an integral role in oxidation and reduction processes. Evidence suggests that iron deficiency may be linked to carcinogenesis [ 11 ]. In contrast, a study of HNSCC showed that those with cervical metastasis had higher serum iron and expression of serum ferritin than those without metastasis [ 12 ]. Malignant tumors may alter calcium metabolism in the bones, intestines, and kidneys through the production of paraneoplastic factors, for instance parathyroid hormone-related protein, leading to bone resorption and the release of calcium into the bloodstream. Furthermore, any metastases to the bones can cause hypercalcemia through the destruction and resorption of osseous tissue [ 13 ]. In Iran, some studies have been conducted on the effect of diet on cancer of the digestive system, but there is no comprehensive study on the assessment of micronutrients in HNSCC patients. Additionally, the lack of clarity and sometimes contradictory results regarding nutrition and trace elements, including copper, iron, zinc, selenium, and calcium, have been reported. Moreover, genetic characteristics and disease patterns vary among different populations. Therefore, this study sought to compare serum levels of essential trace elements between HNSCC patients and healthy individuals. 

## Materials and Method

This case-control study included participants with HNSCC as the case group and healthy individuals as the control group. The case group comprised individuals aged 40–75 years who were newly diagnosed with HNSCC via clinical and histopathological examination at Omid Hospital or Reza Medical Clinic in Mashhad, Iran. Only new cases were selected to ensure that oral cancerous lesions did not influence their nutritional indexes. All participants provided informed consent prior to inclusion. The control group was selected from healthy individuals who visited Mashhad Faculty of Dentistry for dental treatments between June and December 2017. The two groups were matched in terms of sex and age. Exclusion criteria for the case group included uncontrolled diabetes, renal, liver, or gastrointestinal disease, as well as pregnancy and chronic consumption of any supplements or drugs that affect normal nutrition. The exclusion criteria for the control group were the same, with the additional requirement of no history of any cancer.

This case-control study included 40 HNSCC patients and 37 healthy subjects. Three healthy subjects were excluded from the study due to suspicious results from their laboratory tests. Demographic information for both the case and control groups was collected via a checklist. The participants were divided into three age groups: <50 years, 50–70 years, and ≥70 years. Furthermore, based on their body mass index (BMI), they were categorized into four groups: underweight (<18.5), normal weight (18.5–24.9), overweight (25–29.9), and obese (≥30).

The presence or absence of regular daily sports activities was recorded in the questionnaire.

Blood samples of 5 mL were obtained from the median cubital vein and left to clot at room temperature for 10 minutes. Subsequently, the samples were centrifuged at 3600rpm for 5 minutes. Serum iron was assessed using the colorimetric dipyridyl method [ 14 ], copper through the colorimetric oxalyl dihydrazide method [ 14 ], selenium through differential pulse cathodic striping voltammetry [ 14 ], and calcium through the modified orthocresolphthalein complexone method [ 15 ]. Finally, serum zinc was estimated by flame atomic absorption spectrometry [ 16 ].

In this study, the laboratory-provided normal range of serum iron was considered 57-140 µg/dL for females, and 60-160 µg/dL for males. The normal concentration range of calcium in adults was considered to be 8.5-10.5 mg/dL, selenium 87-154 µg/L, copper 70-150 µg/dL, and zinc 70-125 µg/dL.

This study was approved by the Ethical Committee of Mashhad University of Medical Sciences: IR.MUM S sd.REC.1394.72.

Data were entered into SPSS (version 21), and statistical analyses were performed. The Kolmogorov-Smirnov normality test was used to assess the normal distribution of quantitative variables. To compare quantitative data with a normal distribution between two groups, an independent t-test was conducted, and for non-normal distributions, non-parametric tests such as the Mann-Whitney U test were employed. The Chi-square test was used to analyze qualitative variables between two groups. A significance level of 0.05 was adopted for all statistical tests.

## Results

A total of 40 HNSCC patients and 37 healthy subjects were enrolled in this study. The mean age of the HNSCC patients was 62.80±13.029, and the mean age of the control group was 57.92±9.367. Both groups were similarly distributed across the age groups, with 45% of the patients and 56.8% of the controls falling in the 50–70 years age range (*p*= 0.12) (Table 1).

**Table 1:** Demographic properties of case and control groups

Variables		Case	Control	*p* Value
		Number (%)	Number (%)	
Sex	Male	18(45%)	22(59.5%)	*p*= 0.205 *
	Female	22(55%)	15(40.5%)	
Age (years)	˂50	6(15%)	9(24.3%)	*p*= 0.12*
	50-70	18(45%)	21(56.8%)	
	≥70	16(40%)	7(18.9%)	
BMI^¥^	Underweight (˂18.5)	3(7.5%)	0(0%)	*p*˂ 0.001*
	Normal weight (18.5-24.9)	24(60%)	5(13.5%)	
	Overweight (25-29.9)	9(22.5%)	27(73%)	
	Obesity (≥30)	4(10%)	5(13.5%)	
Education	Primary	23(57.5%)	41(0.8%)	*p*˂ 0.001 *
	High school	12(30%)	17(45.9%)	
	Diploma and higher	5(12.5%)	16(43.2%)	
Occupation	Unemployed or retired	2(5%)	10(27%)	*p*= 0.002 *
	Housewife	19(47.5%)	12(32.4%)	
	Labor or farmer	18(45%)	8(21.6%)	
	Employee	1(2.5%)	7(18.9%)	
Marriage	Single	0(0%)	1(2.7%)	*p*= 0.481**
	Married	40(100%)	36(97.3%)	
Smoking	Yes	6(15%)	4(10.8%)	*p*= 0.739
	No	34(85%)	33(89.2%)	
Regular daily sports activities	Yes	4(10%)	22(59.5%)	*p*˂ 0.001*
	No	36(90%)	15(40.5%)	
Systemic diseases	Yes	12(30%)	16(43.2%)	*p*= 0.227 *
	No	28(70%)	21(56.8%)	

*Chi-square test, ** Fisher's exact test, ¥ BMI: Body mass index

The results indicated a significant difference between the two groups based on occupation (*p*= 0.002), education level, BMI, and physical activity (*p*< 0.001 for all variables), but no significant differences were observed regarding other variables, as mentioned in Table 1.

**Figure 1:**
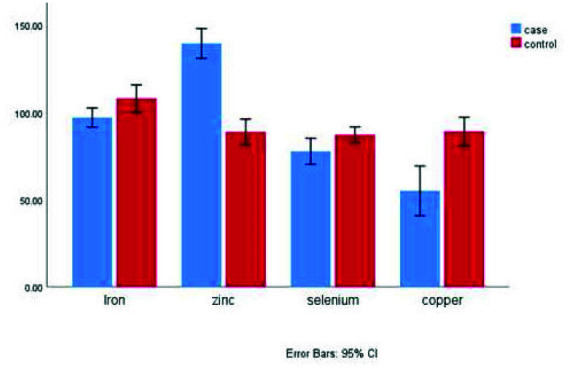
Mean serum concentrations (in µg/dL for Iron, Zinc, and Copper and µg/L for Selenium) of selected trace metal in the case and control groups

Statistical analysis showed that the control group had significantly higher levels of zinc (*p*= 0.006), iron (*p*< 0.001), and selenium (*p*= 0.022) than the case group (Table 2, Figure 1). Conversely, calcium and copper were significantly higher in the case group (*p*< 0.001 for both) (Table 2, Figure 1).

**Table 2:** Mean serum concentrations of trace metals in the case and control groups

Trace metal concentration	Case group (Mean±SD^¥^)	Control group (Mean±SD^¥^)	*p* Value
Selenium	97.10±17.2µg/L	107.97±23.49 µg/L	0.022 *
Copper	139.57±26.70 µg/dL	87.97±17.67 µg/dL	*p*˂ 0.001 **
Zinc	77.15±18.97 µg/dL	87.62±15.58 µg/dL	0.006 **
Iron	61.37±41.29 µg/dL	89.10±24.30 µg/dL	*p*˂ 0.001 **
Calcium	9.84±0.55 mg/dL	9.31±0.46 mg/dL	*p*˂ 0.001 **

* Independent t-test, ** Mann-Whitney U test, ¥ SD: standard deviation

Table 3 demonstrates that 57.5% of cancer patients had low iron levels, compared to 8.1% of control subjects (*p*< 0.001). Additionally, none of the control group had high levels of copper, whereas 25% of case patients did (*p*< 0.001). Moreover, 35% of the case group had low serum zinc levels, in contrast to only 5.4% of the control group (*p*= 0.001). No other variables displayed any significant differences between the two groups (Table 3). Furthermore, a significant association was found between reduced serum iron and zinc levels and the risk of HNSCC (OR=15.33 and OR=9.42, respectively) (Table 3).

**Table 3:** Association of abnormal serum concentrations of trace metals and the risk of head and neck squamous cell carcinoma (HNSCC)

	Case		Control		95% CI^¥^ for OR^≠^	OR^≠^	*p* Value
	n	%	n	%			
Low iron	23	57.5	3	8.1	(4.127,60.578)	15.813	*p*˂ 0.001*
High iron	1	2.5	1	2.7	(0.121,35.139)	2.063	1*
Low selenium	14	35	8	21.6	(0.680,5.224)	1.885	0.194**
High selenium	0	0	1	2.7	-	-	0.481*
Low copper	1	2.5	4	10.8	(0.023,1.987)	0.212	0.189*
High copper	10	25	0	0	-	-	0.001*
Low zinc	14	35	2	5.4	(1.910,43.873)	0.9154	0.001**
High zinc	0	0	1	2.7	-	-	0.481*
Low calcium	0	0	1	2.7	-	-	0.481*
High calcium	6	15	0	0	-	-	0.026*
Systemic disease	12	30	16	43.2	(0.696,4.543)	1.77	0.227**
Smoking	6	15	4	10.8	(0.376,5.632)	1.456	0.739**
Physical activity	4	10	22	59.5	(3.882,44.883)	13.2	*p*˂ 0.001**

*Fisher's exact test, **Chi-square test, ¥ CI: confidence interval, ≠OR: odds ratio

Except for zinc in the control group (*p*= 0.005), no significant differences were observed in mean serum trace metal levels between males and females or between different age groups (Table 4).

**Table 4:** Mean serum concentrations of trace metals according to the age and gender

	Age	(Mean±SD^¥^) control	*p* Value	(Mean±SD^¥^) case	*p* Value
Iron	<50	95±22.68	0.301	72.16±46.5	0.786**
	50-70	85.76±20.84		62.77±44.37	
	≥70	91.57±36.20		55.75±37.39	
Copper	<50	82.66±12.98	0.47	146.83±33.53	0.881**
	50-70	91.95±18.49		141.5±30.5	
	≥70	82.85±19.55		134.68±19.34	
Selenium	<50	0.590	0.590	101.66±18.43	0.573*
	50-70	111.26±26.2		98.38±18.26	
	≥70	106.22±21.62		95.12±14.46	
Zinc	<50	101.66±18.43	0.566	89.33±17.3	0.266**
	50-70	85.42±14.25		71.94±15.47	
	≥70	92±18.36		82.75±20.72	
Calcium	<50	9.24±0.41	0.796	9.24±0.41	0.639**
	50-70	9.36±0.49		9.85±0.54	
	≥70	9.27±0.49		9.75±0.56	
	Gender	(Mean±SD^¥^) control	*p* Value	(Mean±SD^¥^) Case	*p* Value
Iron	Male	89.36±20.42	0.926	57.72±34.86	0.724****
	Female	88.73±29.88		64.36±46.99	
Copper	Male	86.4±16.82	0.369	144.22±22.28	0.118****
	Female	90.26±19.21		135.77±29.81	
Selenium	Male	106.74±27.09	0.705	98.77±20.02	0.582***
	Female	109.78±17.66		95.72±14.68	
Zinc	Male	93.68±14.92	0.005	79±18.04	0.605****
	Female	78.73±12.18		75.63±19.99	
Calcium	Male	9.32±0.46	0.951	9.77±0.54	0.581****
	Female	9.3±0.48		9.9±0.57	

* One way ANOVA for selenium, ** Kruskal-Wallis test for others, *** Independent t-test for selenium, **** Mann-Whitney U test for others,

^¥^SD: standard deviation µg/dL used for Iron, Zinc, and Copper. µg/L used for Selenium. mg/dL used for Calcium

An adjusted logistic regression model was used to assess the correlation of age, sex, smoking, BMI, physical activity, and trace metals with cancer, revealing copper, calcium, and iron concentrations to be significantly different between the two groups (*p*= 0.003, 0.018, and 0.047, respectively) (Table 5).

**Table 5:** Serum concentrations of trace metals in the case and control groups with a forward conditional logistic regression model

	B	*p* Value	Adjusted OR^≠^	95% CI^¥^ for OR^≠^
Copper	-1.82	0.003	0.834	(0.741,0.939)
Iron	0.047	0.047	1.048	(1.001,1.098)
Calcium	-3.74	0.018	0.024	(0.001,0.526)
Constant	51.884	0.004		

* Age, sex, smoking, BMI, physical activity, and trace metals were entered into a forward conditional logistic regression model

^¥^CI: confidence interval ^≠^OR: odds ratio

## Discussion

This study is one of the few case-control studies conducted to assess the concentrations of micronutrients among cancer patients in the Iranian population. Results indicated a significant difference between HNSCC patients and healthy subjects in terms of copper, zinc, calcium, iron, and selenium levels, with serum selenium, zinc, and iron levels being significantly lower in individuals with HNSCC. Conversely, serum copper and calcium levels were higher in the cancer group.

Iron is an essential element for cellular function, and its deficiency may increase the risk of cancer development. Cancer cells require more iron for their activity and express higher levels of transferrin receptors on their surfaces, resulting in lower serum iron concentrations in some cancers [ 11 ], with a decrease in serum iron levels potentially serving as an indicator of cancerous activity [ 17 ]. Iron-binding proteins, such as transferrin and ferritin, provide protection against free radicals; thus, iron deficiency reduces this protective effect and results in increased oxidative stress [ 17 - 18 ]. Ferroptosis, a type of apoptosis, is dependent on the disruption of iron metabolism due to the accumulation of lipid peroxides, with the disruption of iron metabolism being a key factor in some oxidative stress-related conditions, such as malignant diseases [ 19 ]. 

The findings of several studies are in agreement with this research, similarly showing a decrease in the amount of this element in patients with head and neck cancer [ 1 , 14 , 20 ]. In contrast, another study found that salivary iron levels were higher in HNSCC patients compared to the control group [ 21 ]. It appears that an elevated iron concentration could alternatively facilitate carcinogenesis by promoting the production of free radicals via Fenton and Haber-Weiss reactions, thereby inducing DNA damage [ 21 ].

Selenium plays an essential role in the activity of several enzymes, including glutathione peroxidase, iodothyronine deiodinase, metalloproteins, and selenoproteins. As such, it is a key antioxidant, and its deficiency has been linked to nutritional diseases, such as those caused by deficiencies in protein intake, as well as degenerative diseases such as cancer [ 14 ]. 

A number of studies have investigated the effects of selenium on HNSCC, with some suggesting it could possess a protective effect against oral cancer [ 22 - 23 ]. Sonaa *et al*. [ 23 ] recommended selenium supplementation as an adjunct to conventional therapy for oral cancer patients. Other research has reported decreased selenium levels in individuals with oral precancerous or cancerous lesions [ 1 , 14 , 16 ]. Khanna *et al*. [ 14 ] further observed reduced selenium concentrations in early to progressive-stage cancerous lesions, suggesting it may protect against cancer development.

There have been reports of a reduction in zinc levels in pre-malignant conditions as well as cancers of the bladder, prostate, stomach, and gallbladder, and the addition of zinc and other micronutrients to nutritional therapy regimens has been shown to be potentially effective in the treatment of both cancerous and precancerous lesions [ 18 , 20 - 21 , 24 - 25 ]. Zinc acts as a cofactor in transcription processes and helps to maintain homeostasis against tumor progression. It is also actively involved in enzymatic antioxidant systems, such as superoxide dismutase, and may protect DNA from damage and gene mutation. The role of zinc in the production of nucleic acids, scavenging free radicals, and the activity of cytotoxic cells has been demonstrated in the context of oral lesions, and its deficiency has been linked to the development or progression of oral malignant lesions [ 18 ]. Similarly, in this study, serum levels of zinc in cancer patients were lower than in healthy subjects. A cohort study performed in Sri Lanka revealed a higher concentration of some trace metals, including copper and zinc, in the serum of cases compared to healthy controls [ 26 ]. However, in the Sri Lankan study, oral malignant and premalignant lesions were assessed, which may explain the difference between the results of our study and that study. 

In the case of copper, the findings of this and other studies [ 1 , 14 , 17 - 18 , 21 , 24 , 27 ] are in agreement. A relatively smaller number of researchers believe that copper levels in head and neck cancers are reduced, and the ratio of copper to other biomarkers may be a useful indicator of cancer development [ 25 ]. Nevertheless, maintaining copper within the normal range appears to have a protective effect against head and neck cancer [ 28 ]. 

Decreased copper levels reduce the activity of superoxide dismutase, a potent antioxidant [ 28 ]. At the same time, angiogenesis, as well as the proliferation and growth of cancerous tissue, consume copper reserves [ 28 ]. One study found that copper levels in the serum of patients with pre-malignant or malignant lesions of the oral cavity were significantly higher than normal [ 24 ]. 

Higher levels of copper and lower concentrations of calcium were revealed in laryngeal cancer patients compared to the control subjects. In that study, other trace metals were evaluated, and it was concluded that there is an imbalance among the essential and toxic trace metal levels in the serum of laryngeal carcinoma patients compared to the control group. It seems that this imbalance could be effective in the development or progression of the cancer [ 29 ]. Copper has also been shown to be involved in the production of free oxygen metabolites, which through oxidation and regeneration, can create free radicals that bind to cell components, leading to lipid peroxidation, protein oxidation, and nucleic acid damage, potentially promoting carcinogenesis [ 18 ]. 

The finding of increased calcium levels in HNSCC patients in this study aligns with two primary mechanisms explored in the literature: the secretion of substances from the cancerous tissue that induces changes in calcium metabolism in the bones, kidneys, and intestines, or the stimulation of bone resorption in bone metastases [ 13 , 30 ].

This study focused on the simultaneous assessment of multiple micronutrients in newly diagnosed HNSCC patients in Iran, an approach not previously undertaken in similar studies, aiming to identify any correlations between HNSCC, trace metal levels, and other variables, including BMI, education level, occupation, and physical activity. Furthermore, the results of the present study were not affected by the nutritional behaviors of the patients’ post-diagnosis and treatment. However, the main limitation of this research is the incomplete hospital records (such as the stage of the disease and information about subsequent controls) in patients with cancer, as well as the small sample size.

It is recommended that future studies select participants from a variety of geographical regions to better reduce the influence of confounding factors. The findings of the present research are largely consistent with those of similar studies conducted in other countries, suggesting the importance of certain micronutrients, such as selenium, zinc, and iron, in the nutritional regimen. Interventional studies may be designed to investigate whether these micronutrients can be used as supplementary treatments for patients with head and neck cancer. If the results are verified by further research, measuring micronutrient levels could be used as a marker to assess the development or progression of cancer.

## Conclusion

The levels of iron, zinc, and selenium were lower, while the levels of calcium and copper were higher in HNSCC patients compared to healthy subjects. These results suggest that assessing trace metal levels could be beneficial in the screening and evaluation of these patients. We also suggest further investigation of these micronutrients as supplementary treatments for patients with head and neck cancer.
